# Molecular characteristics of diffuse large B-cell lymphoma in the Positron Emission Tomography-Guided Therapy of Aggressive Non-Hodgkin lymphomas (PETAL) trial: correlation with interim PET and outcome

**DOI:** 10.1038/s41408-019-0230-8

**Published:** 2019-08-19

**Authors:** Julia Richter, Andreas Hüttmann, Jan Rekowski, Christine Schmitz, Selina Gärtner, Andreas Rosenwald, Martin-Leo Hansmann, Sylvia Hartmann, Peter Möller, Hans-Heinrich Wacker, Alfred Feller, Christoph Thorns, Stefan Müller, Ulrich Dührsen, Wolfram Klapper

**Affiliations:** 1 Department of Hematopathology, University Hospital Schleswig-Holstein, Kiel, Germany; 2 Department of Hematology, University Hospital Essen, University of Duisburg-Essen, Essen, Germany; 3 Institute for Medical Informatics, Biometry and Epidemiology, University Hospital Essen, University of Duisburg-Essen, Essen, Germany; 4 HTG Molecular Diagnostics Inc, Tucson, AZ 85706 USA; 5 Department of Pathology, University of Würzburg, Würzburg, Germany; 6 Department of Pathology, University Hospital Frankfurt, Frankfurt, Germany; 7 Department of Pathology, University Hospital Ulm, Ulm, Germany; 8 Institut für Hämatopathologie, Kiel, Germany; 9 Hämatopathologie Lübeck, Lübeck, Germany; 10 Department of Pathology, University Hospital Schleswig-Holstein, Lübeck, Germany; 11 Department of Nuclear Medicine, University Hospital Essen, University of Duisburg-Essen, Essen, Germany

**Keywords:** Medical research, Cancer genetics, Translational research

Dear Editor,

Treatment results in diffuse large B-cell lymphoma (DLBCL) are heterogeneous. Established risk models, like the International Prognostic Index (IPI) or molecular features such as MYC translocations and the cell of origin (COO) subtype, are associated with outcome^1^. In the Positron Emission Tomography-Guided Therapy of Aggressive Non-Hodgkin Lymphomas (PETAL) trial, interim positron emission tomography (iPET) after two cycles of rituximab, cyclophosphamide, doxorubicin, vincristine, and prednisone (R-CHOP) has recently been shown to predict outcome independently of the IPI^2^. Whether molecular high-risk features of aggressive B-cell lymphomas are correlated with (and may predict) an unfavorable (positive) iPET result, has not been studied in detail. We aimed to understand the molecular features of DLBCL with a positive iPET by investigating known molecular risk groups, such as *MYC*, *BCL2,* and *BCL6* translocations, and subgroups defined by the COO concept which may benefit from targeted therapies (e.g., activated B-cell-like [ABC] lymphomas).

In the PETAL trial, patients with a positive iPET scan after two cycles of R-CHOP were randomized to receive six additional cycles of R-CHOP or six blocks of an intensive Burkitt’s lymphoma protocol^2^. Patients with a negative scan were continued on R-CHOP. Scans were evaluated using the ΔSUVmax method^3^. A positive interim PET was defined by a decrease of SUVmax at interim PET by ≤66% compared with baseline. A decrease >66% was considered a negative finding ^2^.

Available formalin-fixed paraffin-embedded specimens were analyzed for COO by gene expression using the HTG EdgeSeq System (HTG Molecular Diagnostics, Tucson, AZ, USA). *MYC* and *BCL2* and/or *BCL6* translocations were assessed by fluorescence in situ hybridization (FISH, Vysis-Abbott, Des Plaines, IL, USA). Survival curves for event-free survival (EFS) and overall survival (OS) were compared using hazard ratios (HR) with 95% confidence intervals (CI) from Cox regression and the log-rank test. In addition, we performed multivariable Cox regression analyses for EFS and OS that included COO by gene expression, *MYC* break, “double-hit” status, IPI risk groups (low-risk group includes the IPI risk groups low and low-intermediate, and high-risk group includes the groups high intermediate and high), and iPET result. COO by gene expression profiling was available for 239 patients eight of whom failed the quality control, leaving 231 specimens with gene expression results (Table 1). FISH data were obtained from 253 lymphomas. In 196 cases, FISH for *MYC* and gene expression data were available.

**Table 1:** Molecular features of iPET-negative and iPET-positive DLBCL

	Cell of origin					
	GCB *n* = 102		ABC *n* = 122		Unclass. *n* = 7	
	*n*	%	*n*	%	*n*	%
iPET-negative	90/206	43.7	110/206	53.4	6/206	2.9
iPET-positive	12/25	48	12/25	48	1/25	4
	*p* = 0.6772^a^					

	Translocations									
	MYC				BCL2		BCL6		DH	
	Amp *n* = 48	%	Break *n* = 27	%	Break *n* = 22	%	Break *n* = 37	%	*n* = 16	%
iPET-negative	44/229	19.2	21/229	9.2	18/89	20.2	32/93	34.4	13/227	5.7
iPET-positive	4/24	16.7	6/24	25	4/13	30.8	5/11	45.5	3/22	13.6
	*p* = 1.0000		*p* = 0.0291		*p* = 0.4701		*p* = 0.5153		*p* = 0.1571	

*GE* gene expression, *GCB* germinal center like, *ABC* activated B-cell like, *unclass.* unclassified, *FISH* fluorescence in situ hybridization, *Amp* amplification, *DH* double-hit, *n* indicates number of positive/number of all cases with data

^a^Fisher's exact test GCB versus non-GCB

**Fig. 1:**
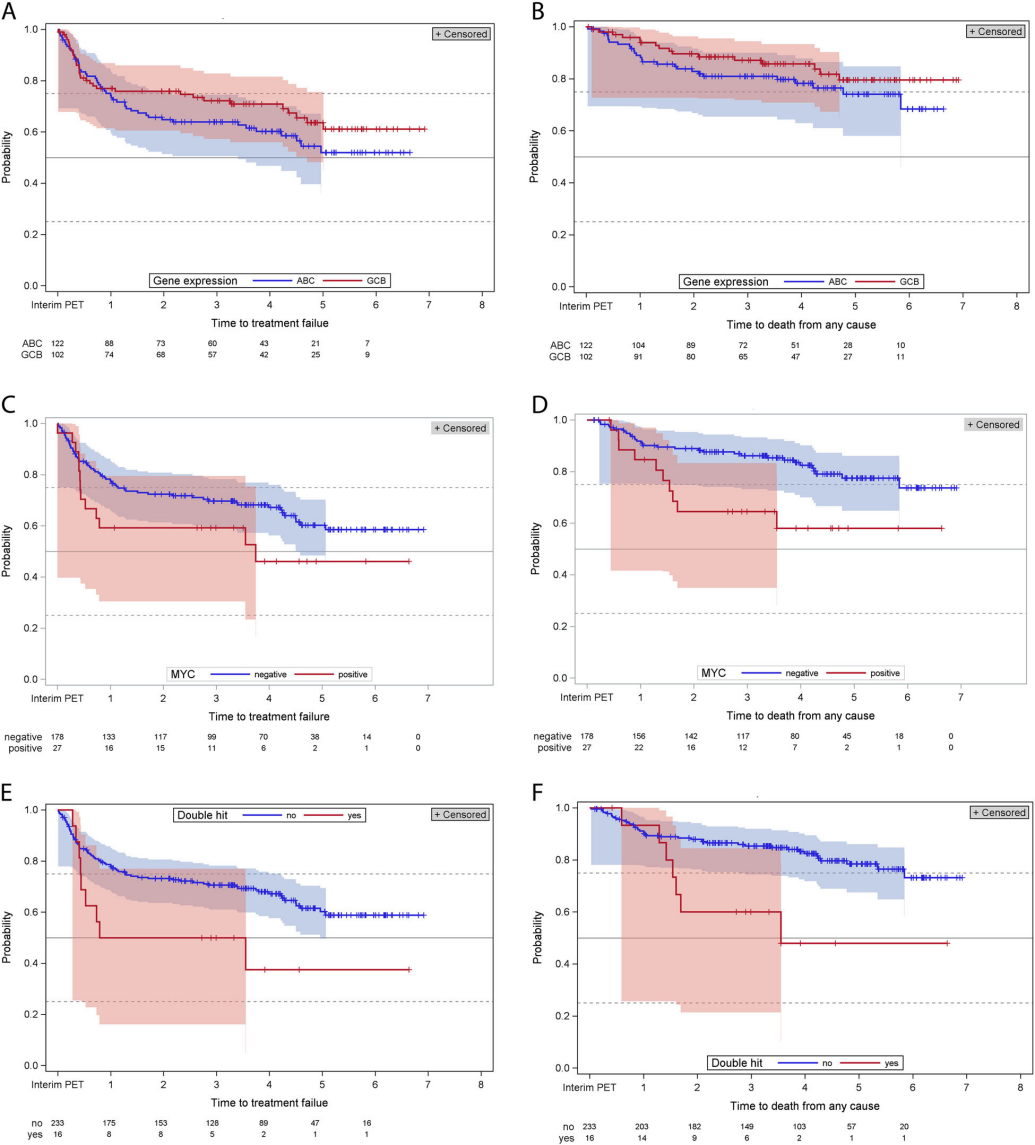
Kaplan–Meier survival analysis for event-free survival (panels **a**, **c**, and **e**) and overall survival (panels **b**, **d**, and **f**) in relation to molecular characteristics of diffuse large B-cell lymphomas treated in the PETAL trial. **a**, **b** Cell of origin as determined by gene expression profiling (**a**, *p* = 0.2077; **b**, *p* = 0.2020). ABC, activated B-cell-like; GCB, germinal center B-cell lymphomas. **c**, **d** MYC-breaks (**c**, *p* = 0.1206; **d**, *p* = 0.0083). **e**, **f** “Double-hit” status (**e**, *p* = 0.0400; **f**, *p* = 0.0049). PET, positron emission tomography

Of 609 DLBCL belonging to the intention-to-treat population of the PETAL trial, 546 (89.7%) were iPET-negative and 63 (10.3%) were iPET-positive^2^. First, we investigated the effect of the molecular parameters on outcome (OS; EFS) in the whole DLBCL cohort, i.e., irrespective of iPET result. Concordance of COO assessment indicated by agreement in classification between immunohistochemistry according to Hans et al.^4^ and gene expression was 83.8%. COO as assessed by gene expression profiling showed no statistical significant association with outcome (*p* = 0.2077 for EFS, *p* = 0.2020 for OS for GCB subtype; Fig. 1a, b). *BCL2* breaks were not associated with outcome (data not shown). By contrast, *BCL6* breaks were associated with decreased survival time (HR 2.105, 95% CI 1.067–4.153, *p* = 0.0282 for EFS; HR 2.783, 95% CI 1.010–7.671, *p* = 0.0388 for OS). An association of *BCL6* breaks with survival has only been shown in one previous study^5^. Recent data suggest that *BCL6* translocations are enriched in the DLBCL category of unclassified COO. They are often associated with other genetic aberrations, such as *NOTCH* mutations^6,7^. In contrast to our observation, the subgroups enriched for BCL6 translocations published so far were characterized by superior survival^6^. A more comprehensive analysis of the mutational landscape of the lymphomas included in the PETAL trial may help resolve this discrepancy.

*MYC* breaks showed a trend for inferior EFS (HR 1.601, 95% CI 0.879–2.915, *p* = 0.1206) and statistically significantly reduced OS (HR 2.531, 95% CI 1.240–5.166, *p* = 0.0083; Fig. 1c, d). A “double-hit“ status (*MYC* translocation and *BCL2* or *BCL6* translocation in the same lymphoma specimen) had previously been shown to be associated with unfavorable outcome^8^. In line with these findings, we observed that “double-hit” was associated with inferior EFS (HR 2.036, 95% CI 1.019–4.068, *p* = 0.0400) and OS (HR 3.006, 95% CI 1.343–6.726, *p* = 0.0049; Fig. 1e, f). In multivariable analysis, only IPI high-risk group compared with low-risk group and positive iPET retained a statistically significant association with EFS (HR 3.828, 95% CI 1.664–8.809, *p* = 0.0016 for IPI; HR 3.326, 95% CI 1.544–7.163, *p* = 0.0021 for iPET) and OS (HR 5.076, 95% CI 1.558–16.532, *p* = 0.0070 for IPI; HR 3.447, 95% CI 1.293–9.190, *p* = 0.0134 for iPET).

In a second step, we assessed the relationship between molecular features and iPET results. The proportion of GCB cases as determined by gene expression profiling was similar in the iPET-negative and iPET-positive groups (90/206, 43.7% and 12/25, 48.0%, respectively, *p* = 0.6772; Table 1). However, lymphomas with a positive iPET scan were significantly enriched for *MYC* translocations (6/25, 24.0%) as compared with iPET-negative lymphomas (21/241, 8.7%, *p* = 0.0394; Table 1). We did not detect a statistically significant difference between iPET-positive and iPET-negative lymphomas with respect to *MYC* amplifications, *BCL2* or *BCL6* breaks, or “double-hit“ status (Table 1).

In a subgroup of 510 DLBCL patients participating in the PETAL trial, we recently confirmed the prognostic impact of baseline total metabolic tumor volume on outcome. Using the 41% maximum standardized uptake value method for measuring tumor volume, the best threshold to distinguish between patients with good versus poor outcome was 328 cm³ (Schmitz et al., submitted). Neither COO nor MYC, BCL2, BCL6 or “double-hit” translocations were associated with tumor volume (data not shown).

In DLBCL, PET scanning provides prognostic information independent of molecular features such as COO or expression of BCL2 and MYC protein^9^. In one small study, a positive iPET was significantly associated with *MYC* translocations^10^. To the best of our knowledge, our study represents the first comprehensive analysis of COO and translocation status in a large prospective trial investigating the value of iPET under controlled conditions. In our study, COO was not associated with outcome as observed in other large prospective trials^5,8,11^. In contrast, lymphomas with MYC translocations with or without additional BCL2 or BCL6 breaks were found to be associated with inferior EFS and OS, confirming the prognostic relevance of this biomarker. In the revised version of the World Health Organization classification of tumors of hematopoietic and lymphoid tissues, which was published after completion of the PETAL trial, “double-hit” lymphomas are separated from DLBCL and classified as high grade B-cell lymphomas with MYC and BCL2 or BCL6 translocations^1^. We confirmed their inferior prognosis in the present investigation. However, the subgroup of “double-hit” lymphomas randomized to receive the Burkitt’s lymphoma protocol was too small (*n* = 3) to investigate the impact of treatment intensification on outcome. Regarding the whole group of iPET-positive patients, intensification of therapy did not improve survival ^2^.

In summary, MYC breaks with or without “double-hit” status were significantly associated with a positive iPET scan. Yet, the unfavorable prognosis of a positive iPET cannot solely be explained by MYC or “double-hit” translocations because most iPET-positive lymphomas lacked these genetic abnormalities. Our results strengthen the role of iPET as a prognostic tool, independent not only of IPI, but also of COO and MYC translocation status. Intensification of conventional chemotherapy failed to improve survival in iPET-positive lymphomas^2^. A more comprehensive molecular characterization of this subgroup may allow us to identify molecular pathways amenable to targeted treatment approaches.
